# An application of advanced oxidation process on industrial crude oily wastewater treatment

**DOI:** 10.1038/s41598-023-29263-y

**Published:** 2023-02-28

**Authors:** Heba A. El-Gawad, Ebrahiem Esmail Ebrahiem, Montaser Y. Ghaly, Ahmed A. Afify, R. M. Mohamed

**Affiliations:** 1 Department of Engineering Mathematics and Physics, Higher Institute of Engineering, El-Shorouk Academy, Cairo, Egypt; 2 Chemical Engineering Department, Faculty of Engineering, Minia University, Minya, Egypt; 3 Chemical Engineering Department, Canal High Institute for Engineering and Technology, Suez, Egypt; 4 Chemical Engineering and Pilot Plant Department, National Research Centre (NRC), Cairo, Egypt

**Keywords:** Environmental sciences, Environmental social sciences, Engineering

## Abstract

Advanced oxidation process, via photo-catalytic oxidation process was demonstrated in this study as one of the promising techniques of simulated oily wastewater treatment. Several effective factors such as initial oil concentration, catalyst dose, stirring speed (rpm), pH value and hydrogen peroxide (H_2_O_2_) dose influencing on the photo-catalytic degradation rate of oily wastewater were investigated. The catalyst used in this work was titanium dioxide (TiO_2_). The solubility of oil in water was increased using emulsifier. Results indicated that the photo-catalytic oxidation process has a good removal percentage of oil from oily wastewater reached to 98.43% at optimum operating parameters of 1 g/L initial oil concentration, 850 rpm, 8 pH, 3 mL H_2_O_2_ and 1.5 g/L of TiO_2_ after 40 min of irradiation time. The degradation reaction follows a first order kinetics with a correlation coefficient (R^2^) of 93.7%. Ultimately, the application of photo-catalytic oxidation processes at these optimum operating parameters on an industrial oily wastewater collected from an effluent stream of Ras Shukair at Red See supplied by Asuit Petrochemical Company was done in Egypt. The results showed that the best oil removal (99%) was achieved after adding 3 mL of H_2_O_2_ in a reaction time of 40 min compared to without adding H_2_O_2_.

## Introduction

Recent increases in human activity at the level of home sectors^1^, agricultural sectors^2^ and municipal sectors^3^ resulting increase in the organic contaminants in wastewater treatment facilities, which causes a discharge to water bodies that does not meet safety and environmental criteria. Due of its hazard, wastewater tainted with oil can actually harm the environment. With the need for more gas stations to service the expanding number of automobiles, the proportion of diesel/gas oil polluted effluent from these facilities has grown as well. Humans who use diesel or gas oil can have skin and eye irritation, however additional impacts have not been well studied^4^. Diesel is a toxicant since it contains polycyclic aromatic hydrocarbons (PAHs), which have the possibility of causing human cancer and are detrimental to human health^5^. For the outflow of oily effluent into surface waters or sewage systems, standards and regulations were created. These laws may differ from one nation to the next and even within one nation.

The necessity for wastewater treatment is dictated by the global increase in the release of slick wastewater, rigorous controls for gushing release, and constant drive for reusing treated wastewater. The selection of treatment methods for smooth wastewater is influenced by factors including wastewater composition, administrative challenges, prices, treatment effectiveness, and wastewater end use^6^. Conventional techniques for treating oily wastewater include dissolved air-flotation, emulsification, chemical coagulation, gravity separation, flocculation, sedimentation, and biological treatment^7^. However, these methods need long settling time, huge land space, and they involve severe sludge handling problems^8^. The produced sludge will need to be treated by novel methods such as adding nanoparticles to produce biogas from sludge^9^ so, according to the quality of natural water and clean water deficiency the development of cheap wastewater treatment and technologies are required.

However, it is challenging to put together a single method or procedure to satisfy the required limitations for outflow because of the complexity of the organic content of oily wastewater. In this regard, researchers are continually working to create a successful therapeutic strategy. Advanced oxidation processes (AOPs), an alternative to traditional treatment methods, have been researched for the treatment of oil-contaminated wastewater. Highly reactive intermediates, such as hydroxyl radicals (HO^**·**^), sulphate radicals, O_2_^–**·**^, H_2_O_2_, and O_3_ are used in AOPs to damage and mineralize the organic contaminants in wastewater by oxidation processes^10^. These oxygen forms are accountable for oxidation and reduction of compounds attached on surface of catalyst. The main principal mechanism in UV-based radical AOP treatment processes is the use ultraviolet light to initiate generation of hydroxyl radicals by direct photolysis of hydrogen peroxide (H_2_O_2_), photo-Fenton reactions or heterogeneous photocatalysis. For the treatment of drinking water and the facilities for reusing water, its technologies have been established and launched. Additionally, scientists and researchers working in the field of the environment are constantly researching a number of AOPs, including those linked to electrochemical processing, electron beam utilization, plasma, microwave, and ultrasound. Such processes include homogenous and heterogeneous processes which may be included by the UV light to enhance the reaction. This UV light may be sourced from natural sun light or from simulated artificial lamp source.The homogenous processes such as Fenton’s reagent, H_2_O_2_ and ozone and heterogeneous photo-catalysis using semiconductors such as TiO_2_, Fe_2_O_3_, CdS, GaP, ZnS and ZnO^11^.

When more biodegradable compound are arranged together or added up to mineralization with carbon dioxide and water, these catalysts cause extremely viable natural matter to deteriorate. The well-known kind of titanium dioxide (TiO_2_) used in photo-catalysis technology. It is the most energetic photo-catalyst (300 nm < 1 < 390 nm) that maintains stability during repeated catalytic cycles. It also exhibits high chemical, physical, and thermal stability, which ensures its widespread and effective use in the treatment of wastewater and water.

In contrast to other water treatment technologies, photo-catalysis has the capacity to perform two purification processes simultaneously: reduction and oxidation. By plating the metals onto the catalyst surface with the help of conduction-band electrons, the reduction is mostly effective for extracting the dissolved metals from water. The dissolved organic contaminant is mineralized using the photo catalyst's oxidation capabilities^12^.

In this study, the heterogeneous photo-catalysis oxidation technology (UV/TiO_2_/H_2_O_2_) was used on a real sample of wastewater for oil mineralization which is coming from an effluent stream of Ras Shukair at Red See supplied by Asuit Petrochemical Company was done in Egypt.The catalyst, which is often a semiconductor, is exposed to radiation in order to produce electron-donor sites (reducing sites) and electron-acceptor sites (oxidising sites), giving redox reagents a lot of potential. The effect of the TiO_2_ catalyst dosage, H_2_O_2_ dosage, stirring speed and initial oil concentration were investigated on this real sample.

## Experimental work

### Experimental set-up

**Figure 1:**
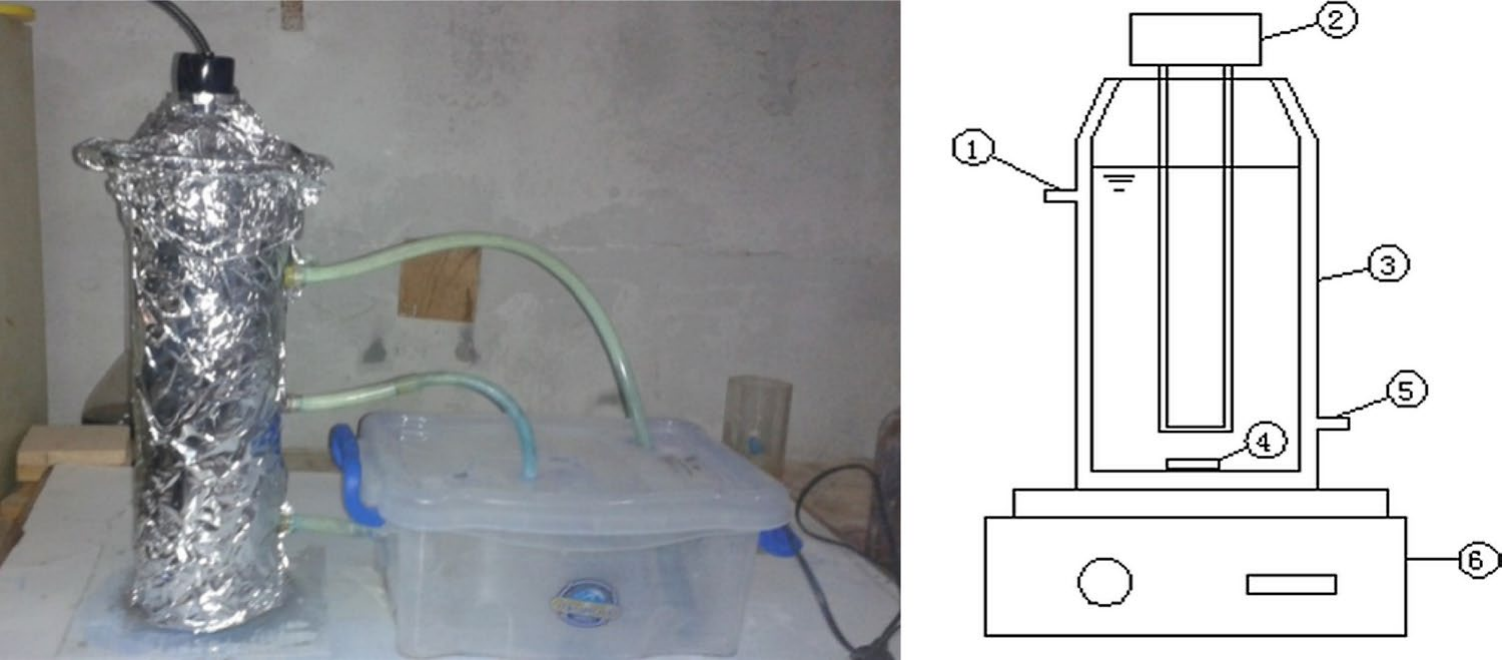
Reactor of UV-based advanced oxidation process: (1) Cooling water out, (2) UV Lamp, (3) Reactor, (4) Stirrer, (5) Cooling water in, and (6) Magnetic stirrer.

In this work, the experiments were conducted in a laboratory-scale reactor in a batch. Experimental set-up used is a closed cycle as shown in Fig. 1. It consists of a plastic tank with 6 L capacity. This tank includes contaminated feed water which will undergo treatment. The tank is linked to the inlet and outlet of a tubular reactor mounted on a hot magnetic stir plate (MSH-300N, BOECO, Germany) to maintain an unchanged composition. This tubular reactor is a precipices UV unit which consists of UV lamp to enhance the reaction and water input and output streams. The long and diameter of tube are 45 cm and 10 cm respectively. UV radiation (254 nm) was generated from UV lamp. Heraeus TNN 15/32, TQ 150 W medium-pressure mercury lamp (Germany) was used as the UV emitter and light source. The lamp was fixed perpendicularly and dipped at the top of the reactor. The lamp was completely dipped in the cylindrical reactor. UV lamp was sheathed in quartz sleeve for protection. The distance between the lamp and the reactor was fixed to be 5 cm to ensure maximum light irradiation.

### Instrumentations

The pump is the first instrumentation used in the experiment for circulation of polluted water through a tubular reactor. It gives output flow rate of 2000 mL/min. The second instrumentation used is centrifuged (DLAB model) to separate TiO_2_ from oil solution to be measured on turbidity meter. The turbidity meter (TU-2016) was used to measure the oil concentration after centrifugation. The sensitive balance (Ohaus Corp Pine Brook, NJ, USA, Item AR 2140) was also used to weigh the number of grams of catalyst. The pH of the sample was measured using a pH meter (model 3505 made by JENWAY Instruments, with a resolution of 0.01 and accuracy of ± 0.02). The pH meter was calibrated on daily basis via buffers of pH 4 and pH 7.

### Reagents

The reagents used in this experiment are distilled water prepared in chemical engineering department laboratories, TiO_2_ catalyst (anatase type) having particle size of 0.005 mm and density of 378 g/cm^3^, diluted H_2_SO_4_ solution, NaOH solution and the oxidant H_2_O_2_ solution used in this experiment were of analytical grade. All these reagents are pure grades delivered from Merck).

### Experimental procedure

The light intensity of lamp (20 W/m^2^ hr) was set up to be nearly firm throughout the experiments. An oil solution was made up in stock solution of 1000 ppm concentration and was subsequently diluted to required concentrations using distilled water. In each experimental run, 5 L of oily wastewater sample with definite oil concentration was placed in the plastic feed tank. A definite dose of the TiO_2_ catalyst was added to the feed tank with strong mixing. Then, the hydrogen peroxide (1–4 mL) was added to the solution. To eliminate the effect of adsorption, the suspension was subjected to mix for about 3–5 min in the absence of UV lamp.

In the UV lamp experiment, the pump was joined to the tank and worked at specified vacuum rate, thus the contaminate solution was circulated from the plastic tank to tubular reactor and back to the plastic tank and the cycle was repeated. The solution was then subjected to stirring via a magnetic stirrer to mix the solution effectively. All the experiments were carried out at ordinary temperature (25 °C).Each experiment lasted 60 min and samples (2 mL each) were drawn from the oil solution at regular intervals (10 min) through the sample valve. Then, the samples were centrifuged to separate TiO_2_ powder. Finally, leave the samples about 10 min to let TiO_2_ settle down. The removal of oil was demonstrated with the reduction in the oily wastewater turbidity.

### Analytical method

**Figure 2:**
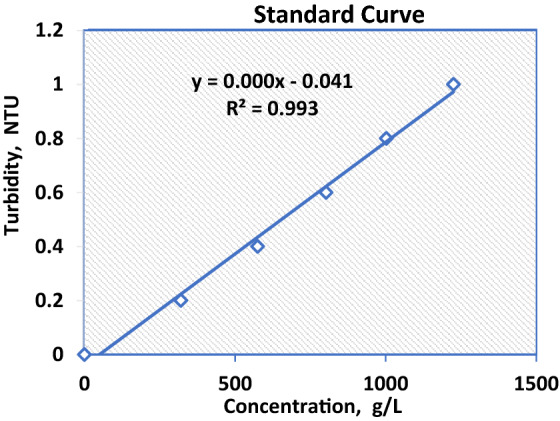
Oil standard calibration curve.

Dilutions of concentrated samples of an oil solution with an initial concentration 1000 ppm is occurred to 10 samples from 1000 to 100 ppm and then the turbidity of each sample was measured. The standard oil calibration curve was made by means of recording the turbidity values for a range of known concentrations of oil solution as shown in Fig. 2. The turbidity readings of different samples after experiments were compared with the calibration curve and converted into concentration (ppm) terms.

### Experimental runs

The oil elimination from wastewater by means of advanced oxidation processes (AOPs) such as photo catalysis oxidation processes was investigated basing on various parameters described on the Table 1. The photo catalysis oxidation process was optimized in a batch-wise experiment to find the optimal factors viz. initial oil concentration, TiO_2_ catalyst dosage, H_2_O_2_ dosage and stirring speed (rpm). The oil elimination efficacy was taking into account through optimization.

**Table 1:** Experimental parameters studied and their ranges.

Parameter studied	Ranges
Initial oil concentration, g/L	0.5–2
TiO_2_ catalyst dose, g/L	0.5–2
H_2_O_2_ dose, mL	1–4
Stirring speed, rpm	650–1000

## Results and discussion

### Influence of pH solution

**Figure 3:**
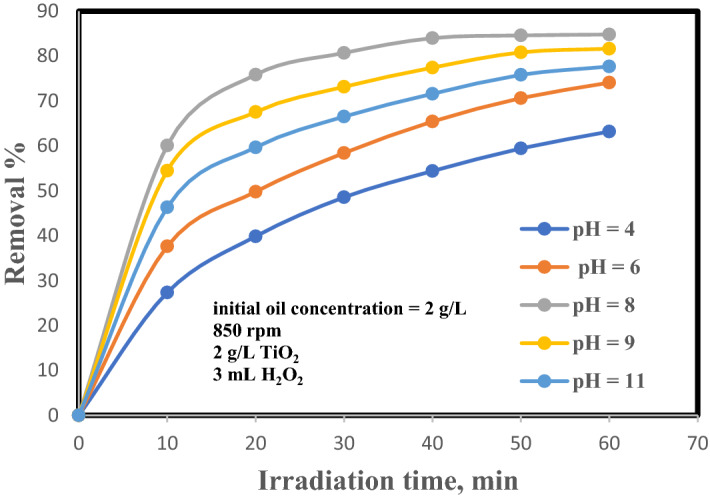
Influence of pH solution on the removal efficiency of oil.

The pH of solution is a significant factor affecting on the photolysis. In order to inspect the influence of pH on photo-catalytic oxidation of the oily solution, a photo-catalytic experiment was achieved with a variety of pH from 4 to 11 in the existence of TiO_2_ at concentration of 2 g/L, H_2_O_2_ = 3 mL, initial oil concentration of 2 g/L, and at 850 rpm. Figure 3 depicts the influence of pH on the elimination percentage. The acquired results elucidated that increasing the pH of the solution up to pH 8 leads to a raised in oil elimination up to 84% at 40 min irradiation time. At lower pH, corrosion of TiO_2_ happened and photo-catalytic reaction was blocked owing to the dissolution of Ti^2+^ in the solution as well as the reduction of oil adsorption over TiO_2_. This leads to a lower of decomposition rate^13, 14^.

The oil removal percentage is increased with pH as OH concentration rise on the TiO_2_ surface in the basic media by photo-induced trapping hole^15^. Oils also consist of the sulphate group in their structure, which are negatively charged in basic conditions (pH > 8). So, efficient oil adsorption is hampered from static electricity causes. Then, at a moderate pH, i.e., between 7 and 8 ideal conditions for oil adsorption and photo production of hydroxyl radicals, obtaining the highest rate of oil elimination versus pH. Moreover, it is obvious that the rate photo-catalytic oxidation rises as the pH of the oily wastewater rises to 8.

### Influence of initial oil concentration

**Figure 4:**
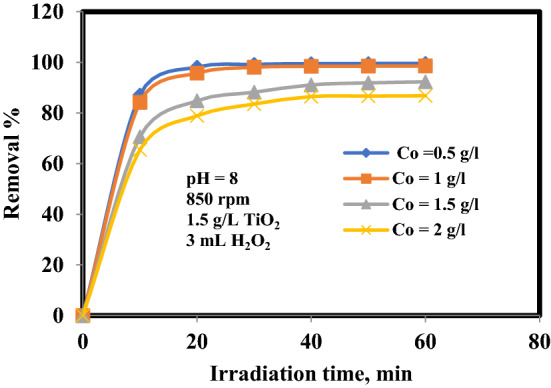
Influence of initial oil concentration on the removal efficiency of oil.

To examine the influence of the initial oil concentration (C_o_) in photo-catalytic process, the various values of initial oil concentration (0.5, 1, 1.5 and 2 g/L) were carried out at 8 pH, H_2_O_2_ = 3 mL, TiO_2_ = 1.5 g/L and 850 rpm. Figure 4 depicts this influence of C_o_ of oil on the elimination percentage. It is obvious that the rise in oil concentration reduce the elimination percentage. Once the oil concentration was raised from 0.5 to 2 g/L, the oil removal was reduced from 99.5 to 86.48% within 40 min of decomposition time. With an increase in C_o_; more and more oil is adsorbed on the TiO_2_ surface. This leads to less generation of OH^**·**^, as there are only fewer active sites for the adsorption of hydroxyl ions and the generation of hydroxyl radicals. Moreover, as C_o_ raised, incident photons were adsorbed by oil before they can reach the catalyst surface. Then, the absorption of photons by the catalyst reduces, and therefore the oil decomposition rate dercreased^12, 16, 17^.

### Influence of TiO_2_ catalyst dose

**Figure 5:**
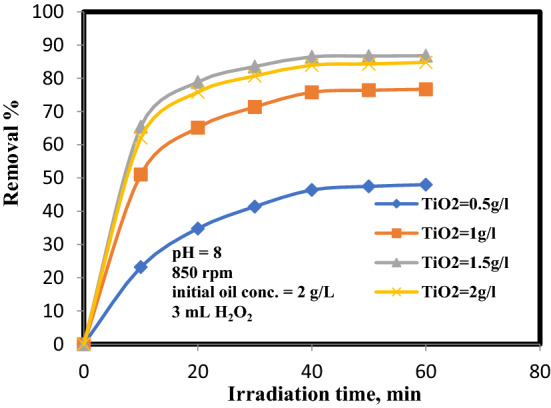
Influence of ttitanium dioxide (TiO_2_) concentrations on oil degradation.

The catalyst capacity influence on oil removal decomposition on TiO_2_ with range of (0.5–2 g/L) was inspected at 8 pH, 850 rpm, initial concentrations of oil and H_2_O_2_ were 2 g/L and 3 mL, respectively. The semiconductor quantity as catalyst is a key factor in heterogeneous photo-catalytic oxidation investigations. It should be noted that, the catalyst capacity affects number of active sites on the photo-catalyst and the permeation of radiation through the suspension^12^. Figure 5 presents that the oil disposal ratio was improved when the catalyst quantity in the reactor elevated up to 2 g/Lover TiO_2_. The raise in disposal percentage was due to the enhancement of OH^•^ generation due to higher TiO_2_ catalytic activity. During the exposure of TiO_2_ to UV light, the positive hole and an electron are produced in the valence band (hv^+^_vb_) and the conduction band (e^−^_cb_). The hydroxyl radicals are produced due to the oxidation of adsorbed water or hydroxyl ions by holes in the valence band on the excited surface as described below^18^. The produced radicals break down the oil particles, raising the disposal rate. Despite this, raising the dose of TiO_2_ beyond an optimum value (1.5 g/L) has a negative impact on the decomposition process. This is a consequence of the fact that overflow TiO_2_ molecules raise the obscurity of the suspension there by reducing the light permeation into the solution, which leads to a decrease in the number of OH^**·**^, and these explications have also been stated in the preceding researches^19, 20^.

1 $${\text{TiO}}_{{2}} + {\text{ hv }} = {\text{ e}}^{ - }_{{{\text{cb}}}} + {\text{ hv}}^{ + }_{{{\text{vb}}}} ,$$

2 $${\text{hv}}^{ + }_{{{\text{vb}}}} + {\text{OH}}^{ - }_{{{\text{surface}}}} = {\text{OH}}^{{\mathbf{ \cdot }}} ,$$

3 $${\text{hv}}^{ + }_{{{\text{vb}}}} + {\text{ H}}_{{2}} {\text{O}}_{{{\text{absorbed}}}} = {\text{OH}}^{{\mathbf{ \cdot }}} + {\text{ H}}^{ + } ,$$

4 $${\text{e}}^{ - }_{{{\text{cb}}}} + {\text{ O}}_{{\text{2 absorbed}}} = {\text{ O}}^{{ - {\mathbf{ \cdot }}}}_{{2}} ,$$

5 $${\text{hv}}^{ + }_{{{\text{vb}}}} + {\text{ oil }} = {\text{ Oxidation}}\,{\text{product,}}$$

6 $${\text{OH}}^{{\mathbf{ \cdot }}} + {\text{ oil }} = {\text{ Degradation}}\,{\text{product,}}$$

7 $${\text{e}}^{ - }_{{{\text{cb}}}} + {\text{oil }} = {\text{ Reduction}}\,{\text{product}}{.}$$

Any further rise in the catalyst concentration leads to a reduction in the oil disposal efficacy which can be illustrated by means of the light scattering generated by the suspended photo resist catalyst^21^. Simultaneously, as a result of the raise in the turbidity of the suspension, there is a reduction in light penetration due to the increase in the scattering effect, and thus the size of the photo-activated suspension reduces^22^. A concentration of 1.5 g/L of TiO_2_ exhibited the bested oil elimination execution. When an excess the TiO_2_ quantity was uptake, the surface area was saturated, then the intensity of light was thus attenuated due to reduced light penetration and raised light scattering^23^.

### Influence of H_2_O_2_ dose

**Figure 6:**
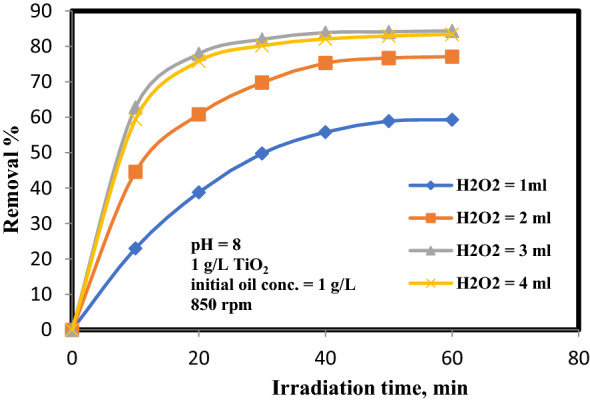
Influence of H_2_O_2_ concentrations on oil decomposition.

The additive of H_2_O_2_ is a significant to improve the photo-catalytic oxidation efficiency since it is used as electron acceptor^24^. Different concentration of H_2_O_2_ (1–4 mL) were added to the solution and irradiated with UV light. Figure 6 displays the influence of H_2_O_2_ on the oil disposal percentage under the operating parameters of oil initial concentration equal 1 g/L, TiO_2_ equal to 1 g/L, 8 pH, and stirring speed of 850 rpm. From this figure, it can be observed that the removal of oil deposits lifted decomposition raised as the concentration of H_2_O_2_ raised from 1 to 3 mL achieving an extreme removal percentage of 83.8% at 3 mL after 40 min of irradiation time. The additive of H_2_O_2_ above this value had a negative effect as mentioned in many previous researches^11, 12, 20^. The reason for this phenomenon is that of hydroxyl radicals produced by the direct photodegradation of hydrogen peroxide are the prime types answerable for the decomposition process. The reason for this phenomenon is that of hydroxyl radicals produced by the direct photo-degradation of hydrogen peroxide are the prime types answerable for the decomposition process. It has been anticipated that rising the H_2_O_2_ concentration minimizing the rate of oil decomposition owing to the reaction of hydrogen peroxide with these radicals producing HO_2_^**·**^. Since HO_2_^**·**^ radicals are not as reactive a HO^**·**^, then low decomposition may acquire, thus acting as an inhibitory agent. Another reason, as H_2_O_2_ concentrations increase, the light intensity decreases, because H_2_O_2_ also soak up lights in the system and leads to lower the degradation efficiency. Increasing the light intensity had a positive effect on photo-degradation and enhanced the reaction rate^25^.

8 $${\text{2H}}_{{2}} {\text{O}}_{{2}} \to {\text{H}}_{{2}} {\text{O }} + {\text{ O}}_{{2}} ,$$

9 $${\text{OH}}^{{\mathbf{ \cdot }}} + {\text{ H}}_{{2}} {\text{O}}_{{2}} \to {\text{HO}}_{{2}}^{{\mathbf{ \cdot }}} + {\text{ H}}_{{2}} {\text{O}}{.}$$

If H_2_O_2_ does is low, HO^•^ forming will also be minimum, which will reduce the treatment efficacy. Therefore, equilibrium must be preserved between surplus and depressed levels of H_2_O_2_^10, 26^.

### Influence of agitation velocity (rpm)

**Figure 7:**
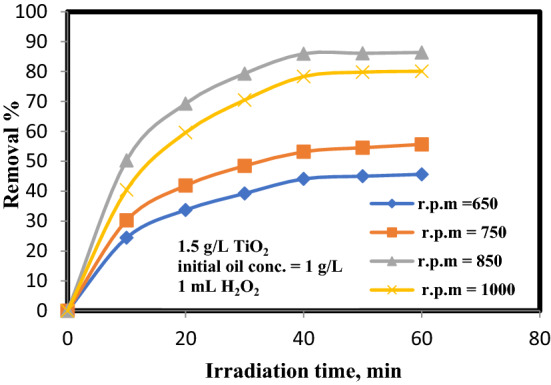
Influence of rotational speed on oil decomposition.

To decide the agitation velocity influence of on oil removal %, tests were executed at C_o_ oil concentration of 1 g/L, 1.5 g/L of TiO_2_, 8 pH, and 1 mL of H_2_O_2_. The final results are demonstrated in Fig. 7 that elucidate that the removal efficiency of oil raises to accomplish a greatest of 85.98% at stirring speed of 850 rpm after 40 min irradiation time after that it tends to drop to 78.36% at 1000 rpm.

### Comparison of the different AOPs

**Figure 8:**
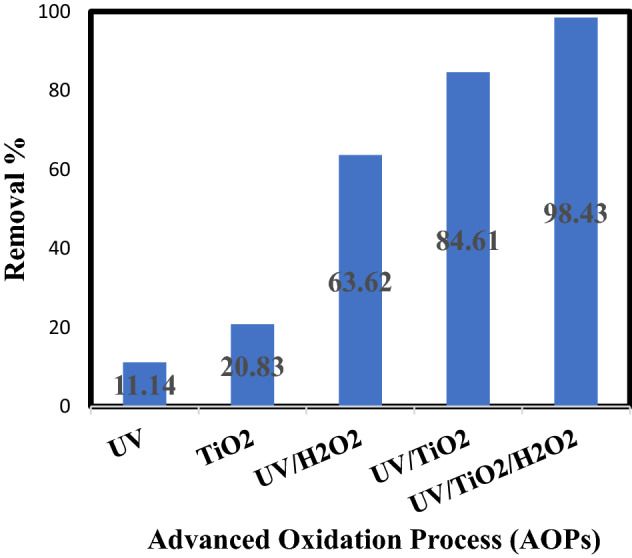
Influence of UV, TiO_2_ photocatalysis, UV/TiO_2_, UV/H_2_O_2_, and TiO_2_/UV/H_2_O_2_, processes on an oil elimination percentage.

Numerous types of AOPs such as UV, TiO_2_ photocatalysis, UV/TiO_2_, UV/H_2_O_2_, and TiO_2_/UV/H_2_O_2_, processes have been inspected for oil decomposition in oily wastewater. A comparison of these different AOPs was made under optimal operating conditions (1 g/L initial oil concentration, 850 rpm, 8 pH, 3 mL H_2_O_2_ and 1.5 g/L of TiO_2_ after 40 min of irradiation time) as elucidated in Fig. 8. The results noted that the TiO_2_/UV/H_2_O_2_ process exhibited the superior oil removal efficiency (98.43%) in oily wastewater. The same figure explain that the addition of H_2_O_2_ to the UV/TiO_2_ process increase obviously the removal percent of oil because H_2_O_2_ plays as electron acceptor. This phenomenon can be elucidated that decomposition of oil is fundamentally controlled by means of the hydroxyl radicals generated by the conjunction of TiO_2_ and H_2_O_2_ in the H_2_O_2_/TiO_2_/UV system. As shown also from the same figure that the percent oil removal in case of TiO_2_ alone (dark process) is occurred due to the effect of TiO_2_ adsorption only. On the other hand, at light photo-assisted processes in the presence of UV light, the oil removal is taken place by the effect of generation of the powerful oxidizing agent (free hydroxyl radicals OH^.^) e.g. (UV/TiO_2_, UV/ H_2_O_2_, UV/TiO_2_/H_2_O_2_ processes). The produced hydroxyl radicals is the main reason for degrading the organic contaminants and the pollutants in the wastewater sample as stated in the literature^27^.

### Photo-catalyst recycle

The lifetime of catalyst is a definitive agent in defining the capacity of the photo-catalytic degradation process for the oily wastewater and for assessing its feasibility in real world purposes. A high-cost reduction can also be got if the photo catalyst can be utilized for a longer period. The catalyst disruption through photo catalytic decomposition can greatly raise the operational cost as a result of the persistent alteration of required catalyst.

The centrifugation process and re-suspension raises the accumulation of the particles and so, decreases the specific surface area. They proposed that the photo catalyst disruption could be relieved by means of superior dispersal or particle-rinsing techniques.

**Figure 9:**
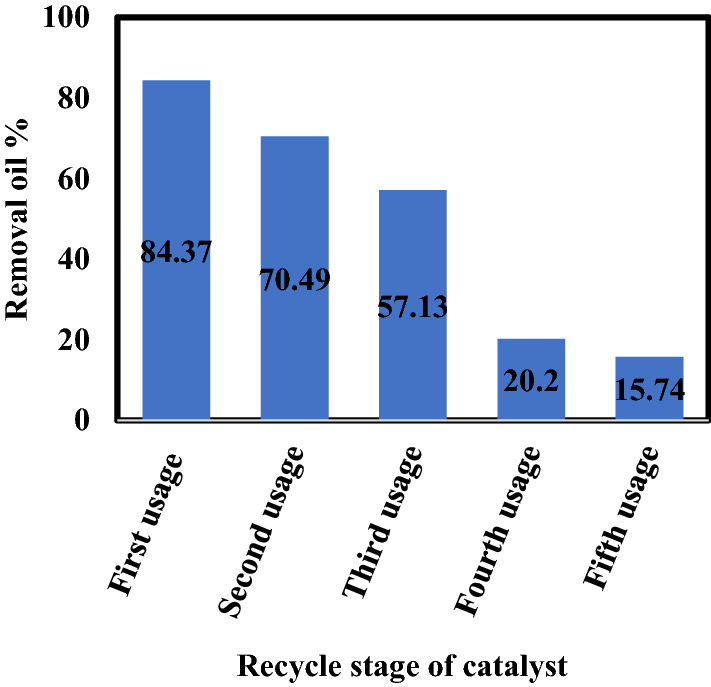
Influence of photo-catalyst recycle on oil removal percentage.

Various catalyst reactivation techniques have been implemented. The Organic contamination of the catalyst can be remedied via calcinations, combustion of organic matter and maintaining the stability and reusability of the catalyst^28,29,30]^. However, this process expends extra energy. Therefore, here the catalyst was recycled 5 times. The catalyst was recovered by rinsing with dilute Hydrochloric acid and water, and there the recyclability was examined. It is noticed that the removal oil percentage decreased to 60%. It was observed that the removal oil percentage decreased to 84.37%, 70.49%, 57.13%, 20.2% and 15.74% upon the 1st, 2nd, 3rd, 4th and 5th photo-catalyst recycling, respectively as clarified in Fig. 9. In our study it is recommended to reuse the catalyst for three times.

## Kinetic studies

**Figure 10:**
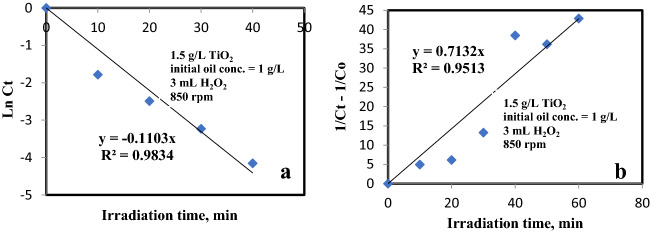
The reaction rate of 1 g/L initial oil concentration at optimum operating parameters. (**a**) First order equation (**b**) Second order equation.

Table 2 clarifies the reaction rate of 1 g/L initial oil concentration, TiO_2_ of 1.5 g/L, 850 rpm and H_2_O_2_ of 3 mL. The linear form of first- and second order kinetic models can be presented in Eqs. (10, 11).

10 $${\text{Ln}}\,{\text{C}}_{{\text{t}}} = {\text{ K}}_{{1}} {\text{t,}}$$

11 $$\frac{1}{Ct}-\frac{1}{Co}={\mathrm{K}}_{2}\mathrm{ t},$$

where C_o_ is the initial concentration of oil and C_t_ is the concentration at irradiation time t, K_1_ and K_2_ are first and second order rate constants in min^−1^ and L g^−1^ min^−1^, respectively, t is the degradation time (in min). A plot ln C_t_ and [$\frac{1}{Ct}-\frac{1}{Co}$] against time for each experiment leads to a straight line whose slope is K_1_ and K_2_, respectively. The regression analysis of the concentration curves against degradation time marks that the rate of reaction can be depicted by means of first order kinetics. The result is shown in Fig. 10.

**Table 2:** The rate of reaction of 1 g/L initial oil concentration.

Reaction rate equation	Kinetic equation	Rate constant	R^2^
First order equation	ln C_t_ = 0.713 t	0.713 min^−1^	0.937
Second order equation	$\frac{1}{Ct}-\frac{1}{Co}=$ 0.11 t	0.11 L min^−1^ g^−1^	0.881

## Case study

**Figure 11:**
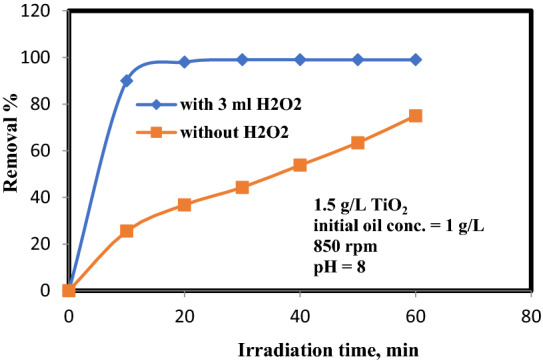
Addition of H_2_O_2_ effect on an oil removal %.

Treatment of an oily wastewater collected from an effluent stream of Ras Shukair at Red See supplied by Asuit Petrochemical Company was investigated. The sewer water from these industries naturally contains fats, oils and greases mixed with a variety of dissolved substances (organic and/or inorganic) in suspension in high concentrations. If they are not treated before being discharged into an open ground or into water streams, it leads to environmental issues and health risks. To avert these types of issues, a process is done for pre-treating oily sewer water. The treatment process was carried out at optimum operating parameters mentioned above (1 g/L of initial oil concentration, TiO_2_ of 1.5 g/L, 8 pH, and 850 rpm). The final results are demonstrated in Fig. 11. These elucidate that the maximum removal % of oil (99%) achieved by adding 3 mL of H_2_O_2_ in a reaction time of 40 min. The oil removal % without adding H_2_O_2_ was 75%. This proved that the addition of H_2_O_2_ is a significant to improve the AOP efficiency.

## Conclusion

Firstly, UV/TiO_2_/H_2_O_2_ was utilized to inspect the AOP performance of oil decomposition in simulated wastewater in the current study. The optimum factors for the process were found to be as follows: 1 g/L of initial oil concentration, TiO_2_ of 1.5 g/L, 850 rpm, 8 pH, and H_2_O_2_ of 3 mL. At these optimum factors, 98.43% of oil was removed from oily wastewater after a reaction time of 40 min. This study found that photo-catalytic oxidation by UV/TiO_2_/H_2_O_2_ is effective in oily wastewater treatment. Secondly, the treatment of oily sewer water collected from an effluent stream of Ras Shukair at Red See supplied by Asuit Petrochemical Company was investigated at these optimum parameters. The maximum removal percentage (99%) was achieved in a reaction time of 40 min with 3 mL H_2_O_2_. By rinsing the catalyst five times in diluted hydrochloric acid and water, the catalyst's recyclability was assessed. According to our studies, the catalyst should be used three times. Thirdly, analysis of oil degradation kinetics was also performed so as to elucidate an optimum operating factors effect on the rate constant of the decomposition reaction. Decomposition reaction follows a first order kinetics with R^2^ of 93.7%.

## Data Availability

Datasets are available in the manuscript. Any additional information and data are available upon reasonable request. The data and materials can be shared with the corresponding author upon reasonable request.
